# Role of ACE1, ACE2, and CCR5-Δ32 Polymorphisms in the Transmission of SARS-CoV-2 to Intimate Contacts

**DOI:** 10.3390/biology14060587

**Published:** 2025-05-22

**Authors:** Maria Pina Dore, Alessandra Errigo, Elettra Merola, Giovanni Mario Pes

**Affiliations:** 1 Dipartimento di Medicina, Chirurgia e Farmacia, University of Sassari, Clinica Medica, Viale San Pietro 8, 07100 Sassari, Italy; mpdore@uniss.it (M.P.D.); gmpes@uniss.it (G.M.P.); 2 Department of Medicine, Baylor College of Medicine, One Baylor Plaza, Houston, TX 77030, USA; 3 Dipartimento di Scienze Biomediche, University of Sassari, Viale San Pietro 43, 07100 Sassari, Italy; a.errigo@studenti.uniss.it; 4 Sardinia Blue Zone Longevity Observatory, 08040 Santa Maria Navarrese, Italy

**Keywords:** SARS-CoV-2, angiotensin-converting enzyme, C-C Chemokine Receptor 5, viral transmission, COVID-19

## Abstract

Background. Despite the high transmissibility of SARS-CoV-2, some individuals remain uninfected despite prolonged exposure to a high viral load, suggesting the involvement of an innate resistance mechanism, possibly underpinned by the host’s genetic factors. The angiotensin-converting enzyme-1 (*ACE1*), *ACE2*, and C-C Chemokine Receptor 5 (*CCR5*) polymorphisms have been shown to influence susceptibility to the infection. In this study, the role of *ACE1*, *ACE2*, and *CCR5* gene polymorphisms in modulating susceptibility to SARS-CoV-2 infection within the context of intimate contact was evaluated. Methods. A cohort of heterosexual couples from Northern Sardinia, characterized by a homogenous genetic background, was recruited during the initial pandemic wave (March–June 2020). In each couple, one partner (index case) tested positive for SARS-CoV-2 by at least two consecutive independent molecular tests (real-time polymerase chain reaction: RT-PCR) on nasopharyngeal swabs. Bed-sharing partners of SARS-CoV-2 positive index cases, resistant and susceptible to the infection, were genotyped for *ACE1* 287 bp *Alu* repeat insertion/deletion (I/D) polymorphism, *ACE2* G8790A (*rs2285666*) variant, and a 32-base pair deletion (Δ32) of *CCR5*. Resistant and susceptible partners to the infection were compared for polymorphisms. Results. Out of 63 couples, 30 partners acquired SARS-CoV-2 infection, while 33 remained uninfected despite intimate exposure. Clinical history was minimal for current or past illnesses. SARS-CoV-2-infected index spouses and partners who acquired the infection developed a mild disease, not requiring hospitalization. The observed distribution of *ACE1* I/D and *ACE2* G8790A genotypes was consistent with previously reported frequencies in Sardinia and across European populations. None of the study participants carried the *CCR5-Δ32* variant. No statistically significant differences (*p* > 0.05) in the allelic or genotypic frequencies of these polymorphisms were observed between the infected and resistant partners. Conclusions. No differences in the distribution of *ACE1*, *ACE2*, and *CCR5* polymorphisms between the two groups were detected. These findings suggest that resistance is likely multifactorial, involving a complex interplay of genetic, immunological, and environmental factors.

## Simple Summary

Some people seem to be resistant to infection by viruses that cause COVID-19, even after close and prolonged contact with someone who is infected. This study explores whether certain inherited genetic traits might explain this phenomenon. Researchers looked at 63 couples in Sardinia, where one partner had COVID-19 during the first wave of the pandemic, and the other either did or did not become infected despite sharing a bed. They focused on specific gene variants that may influence how easily a person becomes infected. The results showed that there were no major differences in these gene variants between the partners who became infected and those who did not. This suggests that resistance to the virus cannot be explained by these genes alone. Other factors (such as how an individual’s immune system works or environmental conditions) are likely to play an important role. Understanding why some people resist infection could help develop better ways to protect the public in future outbreaks.

## 1. Introduction

Coronavirus disease 2019 (COVID-19) was first identified in Wuhan, China, in December 2019 and has since spread rapidly around the world, causing a major global threat to human health, with over 100 million cases and 6.9 × 10^6^ confirmed deaths as of May 2023 [1]. Despite the high transmission efficiency and aggressiveness of the causative agent (SARS-CoV-2 virus), its clinical presentation varies widely, from asymptomatic cases [2] to life-threatening respiratory failure [3]. Transmission primarily occurs through close and prolonged contact with infected individuals who are most contagious during the earlier stages of infection [4]. Remarkably, some people remain uninfected despite repeated and high-level exposure, even in superspreading events [5]. These highly exposed individuals include household members living with recovering SARS-CoV-2 patients [6], partners of those with symptomatic infection who did not undergo quarantine or social distancing [7], healthcare workers in high-risk settings [8], and people exposed to infected biological material without adequate measures [9]. In many cases, they show no symptoms and lack detectable serological markers, suggesting an innate resistance [6]. The reasons behind this resistance still need to be elucidated, but speculatively, good candidate factors are scrupulous compliance with hygiene precautions, a better global state of health, a more efficient innate and adaptive immune system, or even chance. However, in some exposed yet seronegative subjects, the resistance to infection is evident and prolonged over time, making it plausible that the role of protective factors hinges on the host’s genetic makeup.

Like other viruses, SARS-CoV-2 pre-Omicron strains (before September 2020) demonstrated remarkable genetic stability due to the population’s widespread susceptibility and lack of immune pressure. However, subsequent mutations gave rise to variants with increased transmissibility, disease severity, or immune evasion. For example, on the Italian island of Sardinia, wild-type SARS-CoV-2 strains predominantly featured the spike protein variant D614G [10].

SARS-CoV-2’s spike glycoprotein binds to the angiotensin-converting enzyme-2 (*ACE2*) receptor, enabling the virus to enter cells, while the related *ACE1* (angiotensin-converting enzyme-1) has been linked to disease severity [11,12]. The G8790A (*rs2285666*) variant in the *ACE2* gene and a 287 bp *Alu* insertion/deletion (I/D) polymorphism in the *ACE1* gene have been associated with susceptibility to or severity of SARS-CoV-2 infection in various populations [13]. In addition, a 32 bp deletion of the C-C Chemokine Receptor-5 (*CCR5-Δ32*) has been implicated in the host resistance in viral infection [14]. *CCR5* is an essential G protein-coupled receptor on the surface of monocytes, T cells, and macrophages, responsible for driving inflammation in several infectious diseases. The *CCR5–Δ32* variant produces a truncated protein and significantly reduces surface expression of the receptor. Notably, *CCR5-Δ32* is famous for conferring resistance to HIV-1 [15]. Its potential role in COVID-19 has been speculated but remains unconfirmed [14].

Based on these premises, this study examines the role of *ACE1*, *ACE2*, and *CCR5* polymorphisms in determining resistance/susceptibility to SARS-CoV-2 infection, particularly among individuals in close, prolonged contact with infected persons.

## 2. Materials and Methods

### 2.1. Setting

The study was conducted in the urban area of Sassari and the hinterland of Northern Sardinia, Italy. During the initial wave of the COVID-19 pandemic (from March to May/June 2020), the Wuhan-Hu-1 strain (imported from mainland Italy) spread among the population in Northern Sardinia. The Pfizer–BioNTech (New York, NY, USA) vaccine, the first COVID-19 vaccine approved in the United States under Emergency Use Authorization, became available in the Sassari area only starting from late December 2020. According to the Italian National Strategic Plan, early vaccination was prioritized for healthcare workers, followed by individuals classified as fragile, and then the general adult population. Prior to vaccination, the only IgM and IgG antibodies detectable in the blood were those produced following natural exposure to the virus.

### 2.2. Study Population

**Figure 1:**
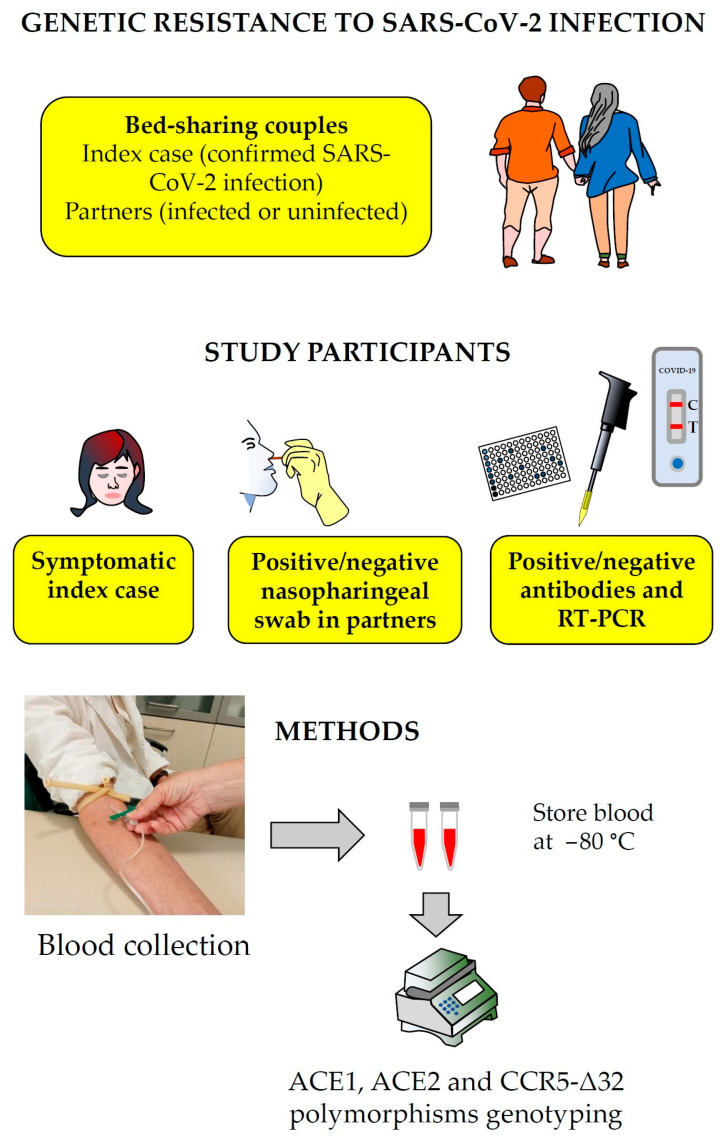
Study design and methodological overview.

Adult bed-sharing heterosexual couples, with or without children, were recruited on a voluntary basis. The inclusion criteria were as follows: (i) one partner had to have tested positive for SARS-CoV-2 (index case), while the other partner had to have remained negative despite prolonged exposure, as confirmed by two independent negative swabs (at 5 and 14 days after the index’s diagnosis); (ii) both partners gave informed consent for genetic testing. All index cases were symptomatic (with flu-like illness) and self-isolated as soon as their COVID-19 diagnosis was confirmed. Recruitment occurred during April–July 2020 through a collaborating COVID-19 testing center. Each couple was classified into two subgroups for analysis: “resistant” partners (exposed but uninfected) and “susceptible” partners (exposed and infected). The term “resistant” is used descriptively for individuals who remained PCR-negative and seronegative after exposure to their infected spouse (Figure 1).

### 2.3. SARS-CoV-2 Status Confirmation

Nasopharyngeal swabs of index cases were collected at the onset of flu-like symptoms (e.g., fever, cough, myalgia, headache, nasal congestion, sneezing, loss of smell or taste) that were strongly suggestive of SARS-CoV-2. Each index case was confirmed SARS-CoV-2-positive by RT-PCR performed by the regional reference lab according to WHO/CDC protocols [16]. Their partners underwent nasopharyngeal swab testing as well; those who consistently tested negative by RT-PCR were classified as resistant.

Qualitative anti-SARS-CoV-2 IgG and IgM antibody detection in whole blood was performed for all partners using the VivaDiag™ SARS-CoV-2 IgG/IgM Rapid Test (Vivacheck, Hangzhou, China), an immunoassay-based lateral flow test. The VivaDiag test demonstrated a sensitivity of 90.6% (95% CI: 84.9–94.4%) and a specificity of 100% (95% CI: 99.4–100%) in validation studies [17,18].

Importantly, the test did not exhibit cross-reactivity with influenza A and B viruses, *Chlamydia pneumoniae*, *Mycoplasma pneumoniae*, or respiratory syncytial virus (RSV) antibodies. Blood IgM and IgG measurements were performed according to the manufacturer’s instructions.

### 2.4. Polymorphism Genotyping

Approximately 10 mL of venous blood was collected from each study participant in EDTA tubes for genomic DNA extraction. DNA was extracted from leukocytes using a standard salting-out procedure within 24 h of collection and resuspended in a TE buffer. Briefly, a lysis buffer (10 mM Tris-HCl, 2 mM EDTA, 400 mM NaCl, 2% SDS) was added to the leukocyte pellets. After Proteinase K digestion at 37 °C overnight and phenol/chloroform extraction, DNA was precipitated with ethanol, washed, and dissolved in TE buffer. DNA samples were stored at –20 °C until analysis. The same extraction protocol was applied to all samples to ensure consistency.

DNA amplification by PCR was conducted in a 25 μL reaction containing ~50 ng genomic DNA, 0.25 μL dNTP mix, 3.75 μL nuclease-free water, and 5 μL TaqMan Universal PCR Master Mix (Thermo Fisher Scientific, Monza, Italy). PCR cycling conditions were optimized for each polymorphism as follows.

The *ACE1*, *ACE2*, and *CCR5* variants were genotyped in all 63 age-matched spouses. The ACE1 I/D gene polymorphism at intron 16 was genotyped by PCR using primers: forward 5′–CTGGAGACCACTCCCATCCTTTCT–3′ and reverse 5′–GATGTGGCCATCACATTCGTCAGAT–3′; followed by a second amplification with the insertion-specific primer 5′–TTTGAGACGGAGTCTCGCTC–30 to avoid misclassification of I/D as D/D due to preferential amplification of the shorter D allele. The *CCR5–Δ32* was genotyped by PCR amplification with primers: forward 5′–CAAAAAGAAGGTCTTCATTACACC–3′ and reverse 5′– CCTGTGCCTCTTCTTCTCATTTCG–3′. The G8790A SNP in ACE2 was genotyped using the forward primer 5′-CATGTGGTCAAAAGGATATCT-3′ and the reverse primer 5′-AAAGTAAGGTTGGCAGACAT-3′. The PCR product (466 bp) was then digested with the enzyme *Hin1II*; the G allele remains uncut (466 bp), whereas the A allele yields two fragments (281 bp and 185 bp). Fragments were resolved on agarose gel to determine genotype.

### 2.5. Statistical Analysis

Statistical analysis was performed using SPSS v22.0 (Chicago, IL, USA). The χ^2^ test (or Fisher’s exact test when appropriate) was used to compare genotype and allele frequencies between groups (resistant vs. infected). A *p*-value < 0.05 was considered statistically significant. We also analyzed the data under dominant and recessive genetic models for each locus. Hardy–Weinberg equilibrium (HWE) for genotype distributions was tested by χ^2^.

### 2.6. Ethical Considerations

Verbal and written informed consent was obtained from each participant. The study was approved by the Independent Ethics Committee of AOU Cagliari, Comitato Etico Indipendente AOU Cagliari (Prot. PG/2021/5418), approved March 2021.

## 3. Results

A total of 63 heterosexual couples were recruited. Among these, 33 partners remained resistant to the infection (19 females) despite prolonged exposure to their infected spouse, while 30 partners tested positive for the infection (15 females) (see Table 1 for cohort characteristics). All participants were of Sardinian (Caucasian) ancestry from Northern Sardinia, a population characterized by a homogenous genetic background [19]. Overall, the study population exhibited minimal comorbidities: four individuals had hypertension, two had celiac disease, two had hypercholesterolemia, two had a history of gastritis, and one had type I diabetes with multiple sclerosis. The remaining subjects had unremarkable medical histories. All infected index spouses and the partners who acquired the infection developed a mild illness with influenza-like symptoms (fever, cough, myalgia, and headache) that did not require hospitalization.

Exposure duration. Couples reported sharing living quarters (and beds) for a range of 3–15 days (resistant group) and 3–12 days (infected group) before isolation was instituted. The mean exposure time was similar: ~8 ± 4 days for resistant partners vs. 7 ± 3 days for infected partners (mean ± SD; difference n.s.). This indicates both groups had comparable periods of intimate contact during the index case’s infectious period.

Serology results. Notably, none of the 33 resistant partners developed detectable anti-SARS-CoV-2 IgM or IgG antibodies, consistent with a complete lack of productive infection. In contrast, all 30 susceptible partners (and all index cases) eventually seroconverted (IgG-positive) following infection. This dichotomy in serological response reinforces the resistant vs. infected classification.

Genotypic analysis: The *ACE1*, *ACE2*, and *CCR5* genotypes for all 63 couples were determined (Table 2). In both resistant and infected groups, the distribution of *ACE1* I/D genotypes (II, ID, and DD) was consistent with the frequencies previously reported in the Sardinian population [20,21,22]. Similarly, the *ACE2* G8790A genotype distribution in our cohort aligned with average frequencies observed in European populations (the A allele being less common in Europe than in Asia). Importantly, the *CCR5-Δ32* variant was not detected in any participant, which is in agreement with its low prevalence (~4% allele frequency) in Sardinia [23].

Comparing resistant vs. infected partners, there were no significant differences in genotype or allele frequencies for *ACE1* or *ACE2* (*p* > 0.05 for all; Table 2). For example, 55% of resistant vs. 50% of infected partners were *ACE1* DD (*p* = 0.78), and 30% of resistant vs. 40% of infected were *ACE2* AA (*p* = 0.11); none of these small differences approached statistical significance. As noted, *CCR5-Δ32* was absent in both groups. All genotype distributions conformed to Hardy–Weinberg equilibrium expectations in each group (p_HWE_ > 0.1 for all loci). Analysis under dominant or recessive genetic models did not reveal any associations either. These results indicate that none of the studied polymorphisms had a detectable effect on whether an exposed individual became infected. No statistically significant differences between resistant and infected partner groups were observed in genotype or allele frequencies (*p* > 0.05 for all comparisons).

## 4. Discussion

Our study aimed to investigate whether certain host genetic polymorphisms (*ACE1* I/D, *ACE2* rs2285666, and *CCR5-Δ32*) confer resistance or susceptibility to SARS-CoV-2 infection in individuals with prolonged intimate exposure to an infected partner. The findings did not reveal significant differences in the frequency of these polymorphisms between resistant individuals and those who acquired the infection. This suggests that these particular genetic variants alone are unlikely to be primary determinants of resistance to SARS-CoV-2 in our cohort. The results support the concept that multiple factors are involved in transmission and resistance.

While SARS-CoV-2 is highly transmissible, a subset of individuals remains uninfected despite high-risk exposures. The role of genetic predisposition in shaping susceptibility and disease severity in viral infections has been widely explored. Notably, a growing body of research suggests that host genetic variations influencing viral entry and immune response may contribute to resistance or susceptibility [24]. However, our data align with other recent studies indicating that common *ACE1*/*ACE2* polymorphisms by themselves are not sufficient to confer protection [25].

*ACE2* serves as the primary receptor for SARS-CoV-2, facilitating viral entry into host cells. Genetic variations in *ACE2*, such as *rs2285666* (G8790A), have been associated with differences in *ACE2* expression and activity, potentially influencing infection susceptibility. For instance, the allele of rs2285666 is linked to higher *ACE2* expression and has been hypothesized to be protective against COVID-19 in some populations. *ACE1*, on the other hand, is part of the same renin–angiotensin system; the I/D polymorphism in *ACE1* has been variably linked to cardiovascular disease risk and inflammation. Accordingly, early in the pandemic, some studies suggested an association with COVID-19 severity (with the DD genotype potentially predisposing to worse outcomes). Our study found no significant difference in *ACE2* or *ACE1* genotype distribution between resistant and infected partners. This is consistent with the lack of associations reported in the literature. For example, a recent review concluded that evidence linking *ACE1* I/D to COVID-19 outcomes is contradictory and likely population-specific [11]. Our findings reinforce that *ACE1/ACE2* polymorphisms, at least the ones studied, do not solely favor the occurrence of the infection, especially within a relatively homogeneous population.

The *CCR5-Δ32* mutation, which results in a truncated and non-functional *CCR5* receptor, is well-known for conferring resistance to HIV infection by preventing viral entry into cells [15]. Early in the pandemic, it was speculated that *CCR5-Δ32* might influence COVID-19 by altering the inflammatory cascade or leukocyte trafficking (since CCR5 binds key chemokines involved in lung inflammation) [14]. One hypothesis was that *CCR5-Δ32* carriers might have reduced severity or susceptibility to SARS-CoV-2 [14]. Our finding that none of the 126 individuals (63 couples) carried Δ32 is not surprising given the known low frequency of this allele in Sardinia [23]. This effectively prevented any direct assessment of its effect, but it underscores that CCR5-Δ32 is too rare in this population to be a significant factor in COVID-19 transmission dynamics. Our results align with larger studies that have not found any meaningful impact of *CCR5-Δ32* on COVID-19 incidence or outcomes in European populations [26].

Considering our negative results for the candidate genes, it is likely that other factors are responsible for the observed resistance in some individuals. Human-to-human transmission is a multifactorial event. Besides the specific polymorphisms examined, differences in innate immune responses or other immune-related genes (such as HLA types or interferon response genes) could play a pivotal role. Recent advances highlight several intriguing possibilities: (i) Pre-existing T cell immunity: some exposed uninfected individuals have been shown to mount a rapid T cell response that aborts infection before it can be established. Swadling et al. reported that individuals with pre-existing cross-reactive memory T cells (notably, CD4+ and CD8+ T cells targeting conserved viral proteins) can experience “abortive” infections that never seroconvert [27]. Such T cell responses, possibly from prior exposure to seasonal coronaviruses, could explain resistance in seronegative people who had contact with the virus; (ii) HLA polymorphisms: variation in HLA genes can affect how well viral peptides are presented to T cells. A recent large study identified HLA-B*15:01 as strongly associated with asymptomatic SARS-CoV-2 infection [28]. Carriers of HLA-B*15:01 were significantly more likely to remain symptom-free, presumably due to effective CD8+ T cell responses against SARS-CoV-2 (including cross-reactive responses to common cold coronaviruses). In general, certain HLA and KIR (killer-cell immunoglobulin-like receptor) combinations in the host might render the immune system particularly adept at early viral control [24]; (iii) Other host factors, including genes involved in the interferon pathway (e.g., *TLR7* in males, or genes identified through GWAS such as *OAS* and *TYK2*), have been implicated in susceptibility [29]. Likewise, non-genetic factors such as viral load of exposure, mucosal immunity, and even the microbiome could influence infection outcomes [30].

It is also worth noting that our study’s couples shared living environments, so behavioral factors were presumably similar at the moment of exposure, although we cannot rule out subtle differences (for instance, one partner might have had closer physical proximity or contact time than the other). Still, the seronegative status of resistant partners indicates a genuine absence of infection despite exposure.

The fact that none of the examined polymorphisms were associated with resistance in our cohort does not diminish the phenomenon of “COVID resistance” but rather indicates that no single common variant in *ACE1*, *ACE2*, or *CCR5* was crucial [29]. For instance, the COVID Human Genetic Effort consortium has been investigating rare genetic variants that might confer resistance, but up to now, no prevalent variant explains the resistant phenotype in most people [31].

Our study presents several strengths, including the well-defined study cohort consisting of heterosexual couples with documented exposure, as well as the genetic homogeneity of the Northern Sardinian population, which reduces confounding factors related to ethnic genetic variability. However, our study is limited by the sample size, making the study’s power less than optimal. We also did not measure quantitative viral load in index cases, which could be a confounding factor in transmission (a resistant partner may simply have been exposed to less virus) [32]. However, all index cases were tested at symptom onset, when viral load is typically high, and all couples cohabited for about a week on average during this phase, suggesting substantial exposure in all cases. Another limitation is that by concentrating on *ACE1*, *ACE2*, and *CCR5*, associations with other important genes might have been missed.

## 5. Conclusions

In summary, among intimately exposed couples in a Sardinian cohort, we observed no difference in *ACE1* I/D, *ACE2* G8790A, or *CCR5-Δ32* genotype frequencies between those who contracted SARS-CoV-2 and those who resisted infection. These findings underscore the importance of exploring broader immunogenetic and environmental factors to understand the intriguing phenomenon of COVID-19 resistance. Identifying what protects these exposed yet uninfected individuals could inform new preventive strategies or therapies.

## Figures and Tables

**Table 1:** Characteristics of the study cohort (index cases and partners).

	Symptomatic Spouses Infected with SARS-CoV-2		
Features	Resistant Partners (n = 33)	Infected Partners (n = 30)	*p*
Sex (F/M)	19 F/14 M	15 F/15 M	0.547
Mean age	48 ± 11 years	48 ± 11 years	
Exposure duration (days)	3–15 (mean 8 ± 4)	3–12 (mean 7 ± 3)	
Comorbidities	5/33 (15%) *	6/30 (20%) *	
IgG/IgM seroconversion	0/33 (0%)	30/30 (100%)	

* Comorbidities were generally mild; no significant group difference (see text for details).

**Table 2:** Genotype and allele frequencies of *ACE1*, *ACE2*, and *CCR5* polymorphisms in partners who did not acquire vs. acquired SARS-CoV-2 infection.

Polymorphism	Genotype	Genotype		*p*-Value	Allele	
		Resistant (n = 33)	Infected (n = 30)		Resistant (n = 33)	Infected (n = 30)
*ACE1*	II	2 (6%)	1 (3%)		I allele: 26% D allele: 74%	I allele: 27% D allele: 73%
	ID	13 (39%)	14 (47%)	*p* = 0.78		
	DD	18 (55%)	15 (50%)			
*ACE2*	GG	7 (21%)	11 (37%)		G allele: 46% A allele: 54%	G allele: 53% A allele: 47%
	GA	16 (49%)	7 (23%)	*p* = 0.11		
	AA	10 (30%)	12 (40%)			
CCR5-Δ32 *	WT/WT	33 (100%)	30 (100%)		Δ32 allele: 0%	Δ32 allele: 0%
	WT/Δ32	0	0			
	Δ32/Δ32	0	0			

* CCR5, Chemokine receptor type 5.

## Data Availability

The original contributions presented in this study are included in the article. Further inquiries can be directed to the corresponding author.

## Abbreviations

The following abbreviations are used in this manuscript:

COVID-19	Coronavirus disease 2019
ACE2	Angiotensin-converting enzyme-2
CCR5	C-C Chemokine Receptor-5
NPs	Nasopharyngeal swabs
RT-PCR	Reverse transcriptase polymerase chain reaction
EDTA	Ethylenediamine tetraacetic acid
HLA	Human leukocyte antigen
GWAS	Genome-wide association study
